# It Takes a Village (or a City): Perceived Economic Strain, Caregiver Ambivalence, and Quality of Life Among Caregiving Grandparents Across Perceived Residential Area Types

**DOI:** 10.3390/healthcare14182972

**Published:** 2026-09-11

**Authors:** Danielle Kristen Nadorff, Margaret Ralston, Laura A. Shillingsburg, Amara L. Mason

**Affiliations:** 1 Department of Psychology, Mississippi State University, Mississippi State, MS 39762, USA; las714@msstate.edu (L.A.S.); alm1491@msstate.edu (A.L.M.); 2 Department of Sociology, Mississippi State University, Mississippi State, MS 39762, USA; mralston@soc.msstate.edu

**Keywords:** caregiving grandparents, economic strain, caregiver burden, caregiver ambivalence, quality of life, perceived residential area type, family stress

## Abstract

**Highlights:**

**What are the main findings?**

- Perceived economic strain showed a strong negative relation with quality of life among caregiving grandparents (*r* = −0.58), and the relation between caregiver ambivalence and quality of life depended on perceived residential area type.
- Caregiver ambivalence was associated with lower quality of life among grandparents who perceived their communities as small towns, and with higher quality of life among those who perceived their communities as urban or major metropolitan areas.

**What are the implications of the main findings?**

- Support programs that target financial strain may matter for caregiving grandparents across settings, and the meaning of ambivalent feelings may differ with the kind of community caregivers believe they live in.
- Because the findings are cross-sectional and drawn from an opt-in online panel, they identify patterns for future research rather than tested intervention effects.

**Abstract:**

Background/Objectives: Grandparent caregiving is a common and demanding role, and perceived economic strain is among the more consistent correlates of caregiver well-being. Guided by Hill’s ABC-X family stress model, this study examined whether perceived economic strain was associated with quality of life (QoL) among U.S. caregiving grandparents through two caregiving appraisals, caregiver burden and caregiver ambivalence, and whether perceived residential area type (a single self-classification item, with higher scores indicating a more urban perception) moderated the ambivalence–QoL relation. Methods: Participants included 424 caregiving grandparents recruited through an opt-in online panel. QoL was measured with the two-item WHOQOL-BREF general facet. Storage codes for the health-satisfaction item were verified against the survey instrument; a scoring error affecting them was corrected, and all results reflect the corrected scoring. A serial mediation model (PROCESS Model 6) and a moderated serial mediation model (PROCESS Model 87) were tested, controlling for age, sex, and marital status entered as categorical indicators, with percentile bootstrap confidence intervals (5000 samples). Results: Perceived economic strain was strongly and negatively associated with QoL (total effect *B* = −4.64, *p* < 0.001; the full QoL regression model explained 38% of the variance, *R*^2^ = 0.38). Greater strain was associated with greater burden, and burden with greater ambivalence, but the hypothesized negative serial pathway was not supported, and the serial indirect relation was positive and small (effect = 0.20, 95% CI [0.04, 0.41]). Perceived area type moderated the ambivalence–QoL relation (interaction *B* = 0.63, *p* = 0.002). Ambivalence was associated with lower QoL among grandparents who perceived their communities as small towns (*B* = −3.97) and with higher QoL among those with urban (*B* = 0.93) or major metropolitan (*B* = 1.09) perceptions. The moderation held across robustness checks, including an ordinal specification of the outcome, but did not replicate with an objective metropolitan versus nonmetropolitan indicator. Conclusions: The findings are cross-sectional, come from a relatively advantaged and nonrepresentative sample, and rest on brief measures, so causal conclusions are not warranted, and practice implications remain preliminary. Even so, the results support the idea that the meaning of caregiving ambivalence for well-being may depend on the kind of community caregivers believe they live in, and the strong relation between perceived economic strain and QoL supports continued attention to financial hardship in policies and programs serving caregiving grandparents.

## 1. Introduction

The number of grandparents providing regular care for their grandchildren is growing [1,2]. These caregiving grandparents range from custodial grandparents, who hold legal responsibility or serve as primary caregivers, to grandparents who provide substantial daily care while a parent remains present, and children may enter their care for many reasons, including parental substance use, incarceration, and child welfare involvement [3]. This growing group warrants attention from healthcare and well-being initiatives, as caregiving grandparents often experience worse health outcomes and higher depression levels than peers who are not caregivers [4]. The relation between specific caregiver stressors, available resources, and personal appraisals is complex and can result in varying impacts on quality of life (QoL). The community context in which caregiving takes place may also matter. Rural settings can present service and access challenges, and they can also offer close ties and community supports [5,6,7]; caregivers’ own perceptions of their communities do not always align with objective geographic classifications [8].

The purpose of this study was to examine whether perceived economic strain was associated with caregiving grandparents’ QoL through two caregiving appraisals, caregiver burden and caregiver ambivalence, and whether perceived residential area type moderated the relation between ambivalence and QoL. Perceived residential area type is a single self-classification item, and we treat it as a contextual moderator rather than as a measure of resources. We organize these variables with Hill’s ABC-X model rather than a general stress-process framework for two reasons. The ABC-X model is explicit that a stressor’s relation to adaptation runs through appraisal, which is what our two appraisal measures are positioned to capture, and it treats the family, rather than the individual worker or patient, as the unit under strain, which matches a caregiving arrangement in which a grandparent absorbs the costs of raising a grandchild. We use it as an organizing framework rather than as a model we test in full, and we state below which components our measures do and do not represent. In the sections that follow, we review the literature on economic strain and grandparent caregiving, describe the guiding theoretical framework, and present the research questions and hypotheses. We note at the outset that the sample was recruited through an opt-in online panel, skews toward suburban and urban perceptions of place, and is more economically advantaged than the custodial grandparent population, so the study addresses variation across perceived community types rather than a specifically rural population.

## 2. Literature Review and Theoretical Framework

### 2.1. Economic Strain and the Well-Being of Caregiving Grandparents

Economic strain is a fundamental challenge for the well-being of caregiving grandparents. Financial stress is associated with depressive symptoms across adults [9,10], and among older adults it is associated with a diminished sense of control and self-worth [11,12]. Longitudinal work suggests that declines in relative household income are associated with a higher risk of depression [13], and stress is a notable risk factor for caregiver burden [14]. These relations may be especially relevant for custodial grandparents. Because the circumstances under which grandchildren enter their grandparents’ care are often sudden and unplanned [3], a sense of lack of control is common, and the added costs of raising a child can worsen it. Custodial grandparenting is also associated with feelings of isolation [15,16]. Rural areas often have fewer healthcare and childcare resources [17]; travel to services is an unexpected expense for many rural caregiving grandparents [18]; and worries about meeting grandchildren’s needs can further erode self-worth [19]. Most of this evidence is observational, so these patterns describe associations rather than established causal effects.

Subjective financial stress (i.e., how people perceive their economic hardship, feelings about their overall financial state, and dissatisfaction with it) tends to relate to mental health and well-being more strongly than objective measures (i.e., actual income, assets, and wealth) [20]. Custodial grandparents may experience both, as this group is at higher risk of lower income [21], and the need to renegotiate income and expenses can heighten subjective financial worries [18,22].

Additional financial pressures are common in custodial grandparenting. As grandparents age, energy and health tend to decline, which can reduce income [21]. To compensate, custodial grandparents often cut back on other expenses, sometimes including their own healthcare. Low-income grandparent caregivers report skipping medication or doctor visits to cover their grandchildren’s expenses [5], and many reassess their finances and adjust the support they provide [18,23,24]. These populations differ from other caregiver groups, and evidence from dementia caregivers or general older samples may not transfer directly to caregiving grandparents, a caution we carry into the discussion.

The need for childcare provided by grandparents is often greater than traditional options can meet, and rural areas typically have fewer childcare resources [17,18]. Grandmothers who provide unpaid care at home often have limited access to respite or support [18,25]. To analyze how economic pressures, caregiving appraisals, and community context relate to grandparent well-being, this study draws on a foundational theory of family stress.

### 2.2. The ABC-X Model of Family Stress

Reuben Hill’s ABC-X model [26,27] explains why some families respond better than others to significant stressors through four components: the stressor event (A), the family’s resources (B), the family’s perception of the stressor (C), and the outcome of crisis or adaptation (X). The model is useful because the components interact. A stressor alone does not determine a crisis, but does so jointly with resources and perceptions [27,28].

The model remains in active use in caregiving research. Choy and colleagues [29] used family stress theory to predict well-being among low-income family caregivers of older adults, and Lin and Silva [30] applied the Double ABC-X model to problematic family caregiving. Alternative frameworks exist for these questions, including stress-process and caregiving-appraisal models, and the present design cannot adjudicate among them. We use the ABC-X model as an organizing framework because of its history in family caregiving research, and we treat our variable assignments as working operationalizations rather than settled mappings.

### 2.3. Applying the ABC-X Model to Caregiving Grandparents

**Figure 1:**
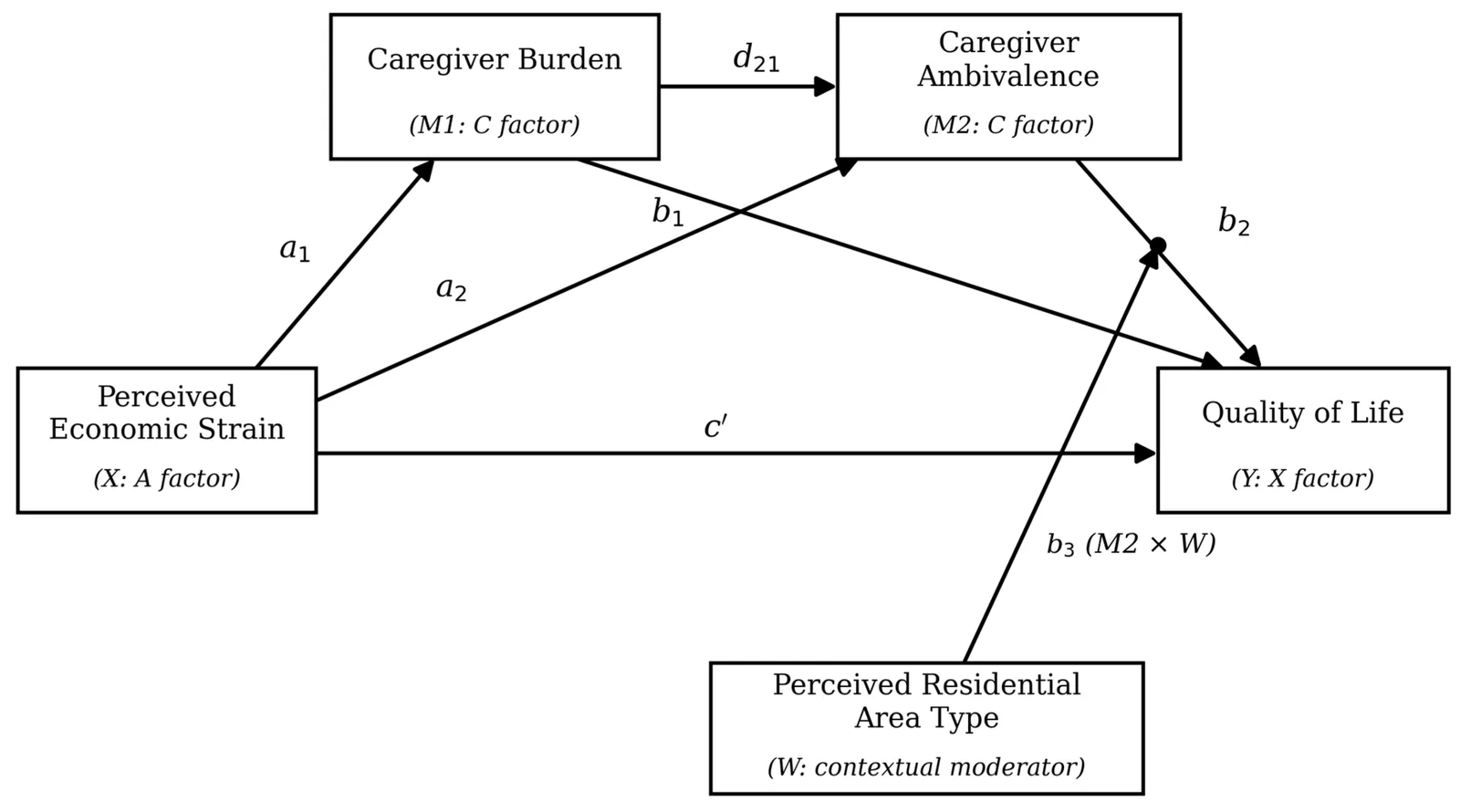
Conceptual model of the moderated serial mediation analysis in caregiving grandparents. Note. This model illustrates the framework guiding the study, adapted from Hill’s (1958) [27] ABC-X model of family stress. The stressor (A: perceived economic strain) relates to the outcome (X: quality of life) through the family’s appraisals of the caregiving situation (C: caregiver burden and caregiver ambivalence, modeled as a serial pathway). Perceived residential area type (W) is modeled as a contextual moderator of the ambivalence–quality of life relation. It is not a measure of resources, so it is not assigned to the B factor, and the B factor is not directly measured in this study. Solid arrows represent regression paths estimated in cross-sectional data (a_1_, a_2_, d_21_, b_1_, b_2_, and the direct path c’), not observed temporal processes. The arrow from perceived residential area type points to the ambivalence–quality of life path to denote moderation (b_3_).

Within the current study, the A factor represents perceived economic strain. Caregiving grandparents often face financial hardship due to the unexpected and prolonged nature of caregiving [31,32], and grandparent-headed households show elevated poverty and food insecurity [5,33]. Consistent with evidence that subjective financial distress relates to health beyond objective conditions [20,34,35], we operationalize the stressor as perceived financial distress rather than as objective economic hardship (see Figure 1).

Family stress applications sometimes treat rurality as part of the B factor. Both conceptually and in our measurement, that mapping is difficult to defend. Rurality is a contextual characteristic rather than a resource, and a single self-classification item cannot represent healthcare availability, transportation, social support, cohesion, stigma, and place identity at once [6,8,36]. We therefore position perceived residential area type as a contextual moderator that may shape how caregiving appraisals relate to adaptation, possibly through differential access to resources, and we leave the B factor unmeasured. Prior work suggests that place conditions family stress processes. Imi [37] found that the effect of cumulative stressors on family functioning differed between rural and urban families.

Rural caregiving research documents both risks and supports. Geographic isolation, service shortages, and stigma can intensify caregiving challenges [6,38], while close community ties and shared values can protect well-being. Heo and colleagues [39] found that rural identity and community bonds buffered the relation between financial stress and life satisfaction among farmers. Rural communities are also heterogeneous, and perceptions of the same place differ across residents [8]. These mixed patterns are one reason we examine perceived community type as a moderator rather than assuming a uniform rural disadvantage.

The C factor comprises caregivers’ appraisals of the caregiving situation, operationalized here as caregiver burden and caregiver ambivalence. Caregiver burden reflects the degree to which caregivers experience their responsibilities as demanding and constraining [40,41], measured here with the Caregiving Burden Scale [42]. Caregiver ambivalence captures the simultaneous presence of positive and negative feelings toward the caregiving role [43,44]. Our rationale for ordering burden before ambivalence is that burden refers to the appraisal of demands, whereas ambivalence refers to an emotional evaluation that, in intergenerational theory, arises when role demands conflict with affection [43,44]. This ordering is theoretical rather than temporal. The two appraisals correlate strongly in this sample (*r* = 0.75), cross-sectional data cannot order them in time, and alternative orderings are plausible; therefore, we report parallel and reversed specifications alongside the serial model. Recent research by Whitley and colleagues [45] used the Double ABC-X model with custodial grandparents during the COVID-19 pandemic, illustrating the continuing use of appraisal constructs in this population.

The X factor is the caregiving grandparent’s level of adaptation. We index adaptation with the general facet of the WHOQOL-BREF, which asks for a global evaluation of quality of life and satisfaction with health [46]. A global evaluation is an appropriate, if partial, indicator of adaptation because it summarizes how the person weighs their life circumstances overall, and quality of life is a common adaptation-relevant outcome in caregiving research [47]. The facet’s two items cannot capture domain-specific functioning, family-level adaptation, or crisis, and we treat this narrowness as a limitation rather than claim the construct represents full family adaptation.

### 2.4. The Present Study

Research on caregiving grandparents documents the relation between economic hardship and well-being [12,18] and the challenges of caregiving in lower-resource areas [5,6,22,23]. What remains less clear is whether caregiving appraisals carry part of the strain-to-well-being relation, and whether the appraisal-to-adaptation relation depends on the kind of community caregivers believe they live in. This study addresses those two questions. The study was not preregistered, and the hypotheses below were specified before the present analyses but after data collection. Because the data are cross-sectional, the mediation models test a theoretically specified ordering of associations rather than causal processes. Specifically, this study addressed the following research questions and hypotheses.

- Research Question 1 (RQ1): Is perceived economic strain associated with quality of life through caregiver burden and caregiver ambivalence?

**Hypothesis 1a** **(H1a).** *Greater perceived economic strain will be associated with greater caregiver burden.*

**Hypothesis 1b** **(H1b).** *Greater caregiver burden will be associated with greater caregiver ambivalence, controlling for economic strain.*

**Hypothesis 1c** **(H1c).** *Greater caregiver ambivalence will be associated with lower quality of life, controlling for economic strain and caregiver burden.*

**Hypothesis 1d** **(H1d).** *There will be a negative serial indirect relation between economic strain and quality of life through greater caregiver burden and then greater caregiver ambivalence.*

- Research Question 2 (RQ2): Does perceived residential area type moderate the relation between caregiver ambivalence and quality of life, and consequently the indirect relation between economic strain and quality of life?

**Hypothesis 2** **(H2).** *The relation between caregiver ambivalence and quality of life will vary across perceived residential area type, as indicated by a significant ambivalence × area type interaction. The conditional serial indirect relations and the index of moderated mediation are reported as secondary, exploratory quantities rather than as part of H2 because they depend on the serial mediation specification.*

## 3. Materials and Methods

### 3.1. Participants and Procedure

Data were collected between March and April 2023 using CloudResearch’s Prime Panels service. Prime Panels aggregates opt-in market research panels comprising millions of individuals and allows targeted recruitment based on specific study criteria [48]. Recruitment methods varied by panel provider and included strategies such as email invitations and listings on a central survey HUB website. Comparative research has shown that data quality from Prime Panels is comparable to that of Amazon’s Mechanical Turk, though Prime Panels participants tend to be more diverse in traits such as age, family structure, religiosity, educational level, and political views [49]. CloudResearch also applies platform-level data quality protections, including screening for duplicate and fraudulent respondents [49,50]. The survey instrument was created using the Qualtrics platform. We estimated its length at 40 to 45 min when contracting with the panel provider. Observed completion times among the retained respondents were shorter and more variable than that estimate, with a median of 29 min and an interquartile range of 20 to 41 min. A survey of this length carries some risk of respondent fatigue.

To be eligible, individuals had to be at least 35 years old, proficient in written and spoken English, identify as a grandparent or step-grandparent to at least one child under 18, and report interacting with a grandchild every day, which a screening question enforced. Eligibility did not require legal custody, co-residence, or a minimum number of care hours, which we describe below and address in the limitations. The instrument embedded three instructed-response attention checks, one each within the caregiving appraisal, parenting, and quality of life blocks (for example, “Please select ‘Very Dissatisfied’”). The first two checks were enforced, and no respondent who failed either one was included in the dataset the panel provider delivered. The third was not enforced, and 27 of the 439 delivered respondents (6.2%) answered it incorrectly, so 412 (93.8%) passed all three. We retained those cases and report a sensitivity analysis excluding them in Section 4.4. Screening records from the survey platform account for all 2565 recorded responses as a sequential flow. Of the 2565 responses, 83 declined consent, 264 reported an age below 35, 322 did not identify as a grandparent or step-grandparent of a child under 18, and 1195 did not report daily interaction with a grandchild, leaving 701 eligible respondents who continued into the main survey. Of these 701, 66 did not complete the survey, 65 completed it but failed an enforced attention check (53 failed the first check and 12 the second, with no overlap), and 131 completed the survey and passed both enforced checks but were not included in the dataset the panel provider delivered, consistent with the platform’s screening for duplicate, fraudulent, and inattentive respondents and its delivery of the contracted number of completes [49,50]. The delivered dataset therefore contained 439 participants. The study received IRB approval from the host institution and adhered to all relevant procedures, including informed consent and confidentiality. After completing the survey, participants were offered informational resources, and compensation followed the terms of the panel through which participants accessed the survey [51].

From the delivered dataset of 439 participants, seven were excluded for missing data on one or more primary study variables, and eight more for missing demographic covariates (age, sex, or marital status), yielding an analytic sample of *N* = 424. Two of the 27 respondents who failed the unenforced attention check were among these 15 exclusions, which is why 25 such respondents appear in Section 4.4 sensitivity analysis. Missingness was limited and scattered across six patterns, affecting age (7 cases), the strain composite (5), and the burden, ambivalence, and sex items (1 each). Included and excluded participants did not differ significantly on age, perceived area type, quality of life, strain, burden, or ambivalence, and Little’s test did not reject the hypothesis that these data are missing completely at random, χ^2^(35) = 45.43, *p* = 0.111 [52]. We therefore used complete-case analysis rather than multiple imputation. With 15 incomplete cases out of 439 (3.4%), imputation would alter the estimates negligibly while adding modeling assumptions, and imputing the outcome or the moderator for cases missing a covariate offers no information gain. Both tests are underpowered against the small number of incomplete cases, so we treat the ignorability assumption as reasonable rather than established. The participants’ average age was 52.75 years (*SD* = 11.64). Most (75.7%) were married, and 64.6% were employed full-time. The grandchildren’s average age was 12.20 years (*SD* = 3.82). Table 1 reports demographic frequencies for the analytic sample.

Because the terms custodial grandparent and caregiving grandparent are sometimes used interchangeably, we describe the caregiving arrangements in this sample explicitly. Eligibility required daily interaction with a grandchild but did not require legal custody or primary caregiving status, so the sample is best described as caregiving grandparents rather than custodial grandparents. Every participant therefore reported daily interaction with a grandchild. Of the full sample, 78.6% lived with the grandchild, and the remaining 21.4% interacted daily without co-residing. Approximately 27% of the full sample lived in skip-generation households, in which no adult child of the grandparent lived in the home. The median reported caregiving time was 30 to 40 h per week, and 54% reported providing 30 or more hours of care per week. Caregiving situations nonetheless range widely, and the absence of a minimum-hours threshold is a limitation. We retain the broader term caregiving grandparents throughout and report sensitivity analyses restricted to co-residing grandparents and to a plausibility-screened subsample in Section 4.4.

The demographic profile of this sample differs in several ways from the national population of custodial grandparents, the closest group for which national benchmarks exist, though our sample was not restricted to custodial grandparents. Compared to national data from the American Community Survey [1], our sample is younger, has a higher employment rate, is less racially diverse, and is more economically secure. Specifically, while the 2023 ACS data show that 46.8% of custodial grandparents are in the labor force, 68.6% of our sample report being employed. Our sample also over-represents White grandparents, includes a slight male majority (51.9%), and is economically advantaged, with 60.6% of the analytic sample reporting household incomes above $100,000 and 55.9% holding postgraduate degrees. The unusually high education and income profile may partly reflect who joins and completes opt-in research panels, and we treat it as a sample characteristic to weigh in interpretation rather than as information about custodial grandparents nationally. We return to this mismatch in the limitations.

### 3.2. Measures

For each measure, we report the number of items, the response scale, the scoring method, an example item, internal consistency in this sample, and distributional characteristics because several measures show floor or ceiling concentration.

Economic Strain: Perceived financial distress was measured with the 8-item InCharge Financial Distress/Financial Well-Being (IFDFW) Scale [53]. Each item uses a ten-point response scale with item-specific anchors. An example item is “How stressed do you feel about your personal finances in general?” (1 = overwhelming stress, 10 = no stress at all). Item responses were averaged and reverse-coded so that higher values indicated greater financial strain (possible range 1–10). Internal consistency in this sample was high (α = 0.93). The average reverse-coded score was 3.83 (*SD* = 2.15), with a right-skewed distribution (skewness = 0.93) and 5.7% of the analytic sample at the scale floor. Scores correlated −0.55 with household income category (Spearman ρ = −0.47), supporting convergent validity. We emphasize that the IFDFW captures subjective financial distress rather than objective financial conditions, and we therefore describe the construct as perceived economic strain throughout.

Perceived Residential Area Type: Participants’ perception of their living environment was assessed with a single item: “Which of the following best describes the type of area in which you live?” Response options were 1 = very rural, 2 = small town, 3 = suburban area, 4 = urban area, and 5 = major metropolitan area, so higher scores indicate a more urban perception. In the analytic sample, 36 participants selected very rural, 29 small town, 85 suburban, 215 urban, and 59 major metropolitan (*M* = 3.55, *SD* = 1.08). We refer to the measure as perceived residential area type because the item asks for a self-classification of place, and we describe direction in terms of perceived urbanicity. A single ordinal item of this kind mixes settlement size, density, and identity; its categories cannot be assumed equally spaced, and reliability cannot be estimated for one item, so we analyze it both as a continuous moderator and categorically (Section 4.3) and treat it strictly as a summary of perceived place. A sensitivity analysis using an objective rurality indicator appears in Section 4.4.

Caregiver Ambivalence: Ambivalence in the caregiving role was measured using an adapted version of the Caregiving Ambivalence Scale (CAS), initially developed by Losada and colleagues [54] and later applied to broader caregiving contexts [55]. The CAS assesses the simultaneous presence of positive and negative emotions experienced by family caregivers. The scale includes 5 items, each with four response options scored 0 (never) to 3 (always), yielding a total score from 0 to 15. An example item is “I have at the same time positive and negative feelings toward [grandchild].” For this study, the word “relative” in each item was replaced with the name of the grandchild with whom the participant spent the most time. This adaptation has not been independently validated, which we note as a limitation. Internal consistency in this sample was good (α = 0.88). The mean CAS score was 4.78 (*SD* = 4.52), with 23.3% of the analytic sample at the scale floor. The CAS correlates 0.75 with the burden measure described next, so the two appraisals overlap substantially, and their unique coefficients should be interpreted with that overlap in mind.

Caregiver Burden: Caregiver burden was measured using the Caregiving Burden Scale (CBS) [42]. The version administered in this study includes 13 statements about the demands of caregiving, each answered with a dichotomous response indicating whether the participant disagreed (0) or was neutral or agreed (1). The total is therefore a count of endorsed burdens from 0 to 13, and coefficients involving the CBS are interpreted per burden endorsed. The dichotomous format discards response gradations, and combining neutral with agreement means the count reflects non-disagreement rather than strict endorsement, both of which we note as limitations. An example item is “I never feel free from the care for [grandchild].” Internal consistency in this sample was high (Kuder–Richardson 20 = 0.94), which for closely related items can partly reflect item redundancy. The mean count was 4.93 (*SD* = 4.60), with 7.8% of the analytic sample at the floor and 14.2% at the ceiling. Because the CBS measures perceived burden rather than the amount, frequency, or time demand of care provided, we treat this construct as an appraisal of caregiving demands rather than as a measure of caregiving intensity.

Quality of Life: Overall quality of life was assessed using the general facet of the World Health Organization Quality of Life—Brief (WHOQOL-BREF) instrument [46,56]. This facet consists of two items, “How would you rate your quality of life?” and “How satisfied are you with your health?”, each rated on a five-point scale. During data verification, the storage codes for the health-satisfaction item were checked against the survey instrument. The item’s five response options had been stored with noncontiguous codes, and an earlier recode had assigned the highest option to the wrong scale point. The corrected mapping was checked for convergent plausibility against other health indicators collected in the same survey (SF-12 physical and mental component scores, self-rated general health, and depressive symptoms), each of which changes monotonically across the corrected item, and all analyses in this article use the corrected scoring. Table S1 reports the full audit trail for the affected item (response label, stored code, previously assigned value, and corrected value for each response option). Following the WHOQOL scoring manual, the two items were summed and transformed to a 0–100 metric [56]. The two items correlated at 0.61 (Spearman–Brown reliability = 0.75). The mean transformed score was 78.80 (*SD* = 20.41), with a left-skewed distribution (skewness = −1.14), nine observed score values, and 27.8% of the analytic sample at the scale ceiling. We used the general facet because the study’s outcome of interest was global quality of life. It does not capture domain-specific functioning, and we note its brevity and coarseness as limitations.

### 3.3. Data Analysis Strategy

Primary analyses were conducted using IBM SPSS Statistics (Version 29.0.2.0) and the PROCESS macro [57], and all reported coefficients were reproduced from the raw data by the research team in StataNow/MP 19.5 (StataCorp LLC, College Station, TX, USA) as a cross-software verification, which was also used for the diagnostic and sensitivity analyses described below. In all models, analyses controlled for grandparent age, sex, and marital status. Because sex and marital status are nominal, both were entered as indicator variables: female versus male (male as the reference category), and widowed, divorced, single, never married, and other versus married (married as the reference category). One analytic-sample participant selected a not-listed sex option. That participant was retained and carries a separate indicator, so no sex value was imputed for them, and the interaction reported below is unchanged when this case is excluded instead (*B* = 0.63, *p* = 0.002). In the correlation table reported in Section 4.1, marital status appears as a married versus not married indicator, and sex as a female versus male indicator computed on the 423 participants who selected male or female. Indirect relations were assessed using percentile bootstrap confidence intervals based on 5000 samples [57]. To address RQ1, a serial mediation model was tested with PROCESS Model 6, and to address RQ2, PROCESS Model 87 added the ambivalence × area type interaction. Conditional relations were probed at each observed value of the moderator, and because equal spacing between the five categories cannot be assumed, the moderator was additionally treated categorically. Mediation models estimated on cross-sectional data cannot establish temporal ordering, so parallel and reversed-order specifications are reported alongside the serial model [58], and specific indirect paths are interpreted as decompositions of association rather than as processes. Regression diagnostics included variance inflation factors, heteroscedasticity-robust (HC3) standard errors, and influential-case checks, and a Harman single-factor test probed common-method variance [59]. We did not adjust for multiple comparisons across the primary and sensitivity models, so the overall pattern of results, rather than any single interval, should carry the interpretive weight.

Five additional analyses probed the robustness of the primary results: (a) re-estimating both models with an expanded covariate set (education, household income, employment status, and co-residence with the grandchild); (b) replacing the perceived moderator with an objective metropolitan versus nonmetropolitan indicator derived from rural–urban commuting area (RUCA) codes [60] matched to participants’ ZIP codes; (c) re-estimating both models within the co-residing subsample; (d) re-estimating both models within a plausibility-screened subsample that excluded caregiver–grandchild age gaps under 26 years, grandchildren under age 3, and cases with missing ages; and (e) re-estimating the two focal associations, the strain relation and the ambivalence × area type interaction, with a proportional-odds (ordered logistic) specification that treats the nine observed outcome values as ordinal categories. A sensitivity power analysis indicated that, with *N* = 424 and α = 0.05, the design provided 0.80 power to detect interaction effects as small as *f*^2^ = 0.019.

## 4. Results

### 4.1. Descriptive Statistics

Descriptive statistics for the primary study variables, calculated on the analytic sample of 424 participants, are presented in Table 2. Perceived strain averaged 3.83 (*SD* = 2.15) on the 1 to 10 metric. Quality of life averaged 78.80 (*SD* = 20.41) on the corrected 0–100 general facet. The burden count averaged 4.93 (*SD* = 4.60), and ambivalence averaged 4.78 (*SD* = 4.52). The distribution of perceived residential area type appears in Section 3.2. Because validated benchmarks are not available for these measures in this population, we describe distributions rather than interpret score levels against thresholds. Taken together, the distributions describe a sample that is, on average, relatively advantaged, a point we address in the discussion.

Bivariate correlations (Pearson *r*) were examined among the primary study variables and demographic covariates in the analytic sample (see Table 3). Perceived economic strain showed a strong negative relation with quality of life (*r* = −0.58, *p* < 0.001) and was negatively correlated with perceived urbanicity (*r* = −0.39, *p* < 0.001). Quality of life was positively correlated with perceived urbanicity (*r* = 0.34, *p* < 0.001). Caregiver burden and caregiver ambivalence were strongly correlated (*r* = 0.75, *p* < 0.001). At the bivariate level, burden was not correlated with quality of life (*r* = 0.04, *p* = 0.452), and ambivalence was modestly and positively correlated with quality of life (*r* = 0.17, *p* < 0.001). These bivariate relations differ from the adjusted paths reported below, so the mediation decomposition reflects conditional relations that emerge when strain and both appraisals are modeled together, and we interpret it with that in mind.

### 4.2. Research Question 1 Results

To address Research Question 1, a serial mediation analysis was conducted. Perceived economic strain was the independent variable (X), caregiver burden (CBS) was the first mediator (M1), caregiver ambivalence (CAS) was the second mediator (M2), and quality of life was the dependent variable (Y). Full results, including covariates, total effects, and standardized estimates, are presented in Table 4.

Consistent with Hypothesis 1a, greater perceived strain was associated with a higher burden count (*B* = 0.40, *SE* = 0.11, *t*(415) = 3.50, *p* = 0.0005, about 0.4 additional endorsed burdens per strain point). The burden model was significant, *R*^2^ = 0.11, *F*(8, 415) = 6.29, *p* < 0.001. Supporting Hypothesis 1b, greater burden was associated with greater ambivalence (*B* = 0.69, *SE* = 0.03, *t*(414) = 21.22, *p* < 0.001), with no direct strain-ambivalence relation (*B* = −0.01, *p* = 0.889). This model explained substantial variance in ambivalence, *R*^2^ = 0.59, *F*(9, 414) = 66.20, *p* < 0.001.

In the model predicting quality of life, *R*^2^ = 0.38, *F*(10, 413) = 25.52, *p* < 0.001. Perceived strain retained a strong negative direct relation with quality of life (*B* = −4.64, *SE* = 0.43, *t*(413) = −10.88, *p* < 0.001), and the total effect was of the same size (*B* = −4.64, *p* < 0.001; standardized total effect = −0.49). Contrary to Hypothesis 1c, ambivalence was positively associated with quality of life when strain and burden were controlled (*B* = 0.72, *SE* = 0.27, *t*(413) = 2.63, *p* = 0.009), and the burden count was not significantly associated with quality of life (*B* = −0.48, *SE* = 0.26, *t*(413) = −1.84, *p* = 0.067).

The hypothesized negative serial indirect relation was not supported. The serial path (strain → burden → ambivalence → quality of life) was positive and small (effect = 0.20, 95% CI [0.04, 0.41]; standardized = 0.02), and the specific indirect paths through burden alone (−0.19, 95% CI [−0.43, 0.01]) and through ambivalence alone (−0.01, 95% CI [−0.12, 0.13]) were not significant. Therefore, H1d was not supported. The standardized magnitudes indicate that these indirect decompositions carry almost none of the strain–quality of life relation, which travels through the direct path. Given the strong burden–ambivalence correlation, the difference in sign between the adjusted and bivariate ambivalence relations, and the sign changes across the specifications reported in Section 4.4, we treat the indirect-path pattern as a fragile decomposition of overlapping appraisals rather than as evidence of a caregiving process [61].

### 4.3. Moderation Results: Research Question 2

To address Research Question 2, PROCESS Model 87 added the ambivalence × perceived area type interaction. The overall model was significant, *R*^2^ = 0.40, *F*(12, 411) = 22.84, *p* < 0.001. Perceived strain retained its negative relation with quality of life (*B* = −4.30, *SE* = 0.44, *t* = −9.68, *p* < 0.001). The main effect of area type was not significant (*B* = −0.59, *SE* = 1.08, *t* = −0.55, *p* = 0.586).

**Figure 2:**
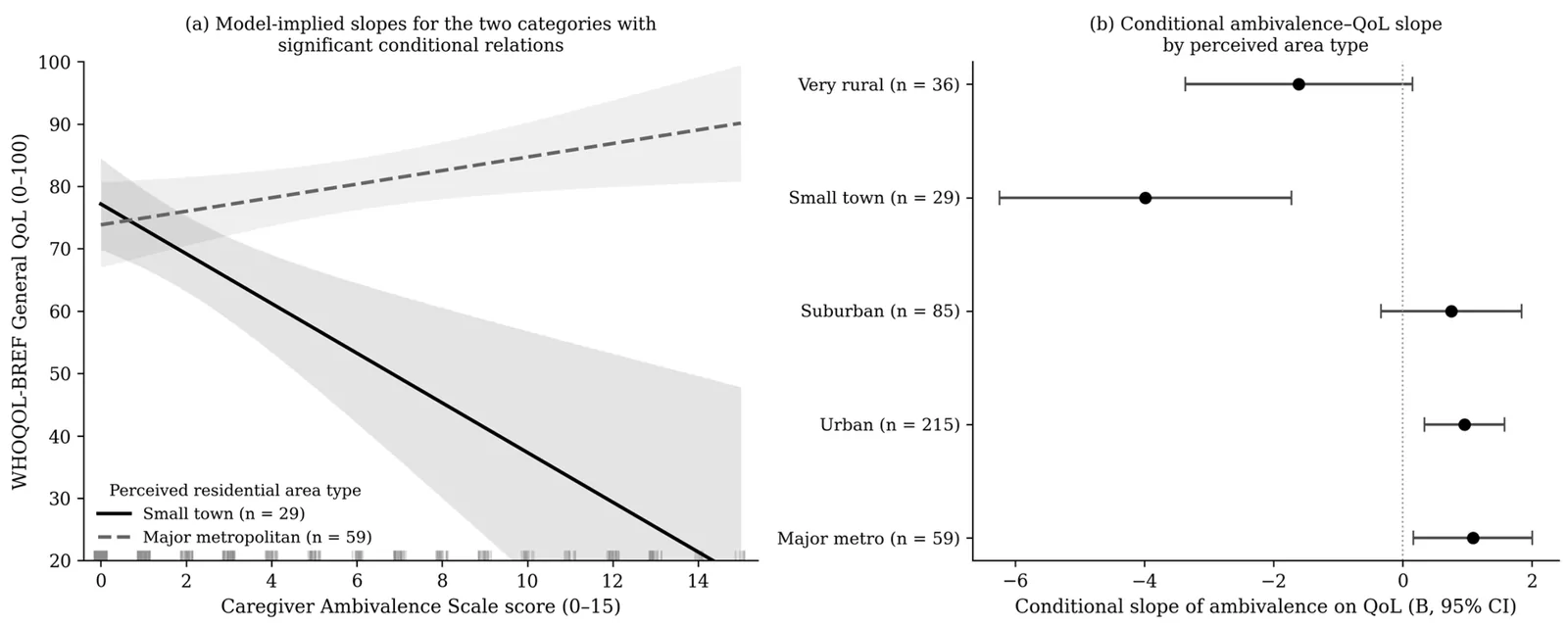
Conditional relations between caregiver ambivalence and quality of life across perceived residential area type. Note. Panel (**a**) shows model-implied simple slopes from the categorical moderator model for two illustrative categories with contrasting significant conditional relations, small town (solid) and major metropolitan (dashed), with all other predictors held at sample means. The urban category also shows a significant positive conditional relation, and Panel (**b**) reports every category. Shaded regions are 95% confidence bands, and tick marks along the horizontal axis show the observed ambivalence distribution. Panel (**b**) shows the conditional slope of ambivalence on quality of life for every perceived area type with 95% confidence intervals and category sample sizes. Quality of life uses the corrected scoring described in Section 3.2.

Supporting Hypothesis 2, the interaction was significant (*B* = 0.63, *SE* = 0.20, *t* = 3.11, *p* = 0.002; Δ*R*^2^ = 0.014, *F*(1, 411) = 9.66, *p* = 0.002). Because equal spacing between the five categories cannot be assumed, the moderator was also treated categorically. The categorical interaction was significant, *F*(4, 405) = 6.14, *p* < 0.001, and fit better than the linear coding, *F*(6, 405) = 3.15, *p* = 0.005, so we emphasize the category-specific results. Ambivalence was associated with lower quality of life among participants who perceived their communities as small towns (*B* = −3.97, *SE* = 1.15, *p* = 0.0007), with a same-direction relation among very rural perceivers that did not reach significance (*B* = −1.51, *SE* = 0.90, *p* = 0.095, *n* = 36). Ambivalence was associated with higher quality of life among urban (*B* = 0.93, *SE* = 0.32, *p* = 0.004) and major metropolitan (*B* = 1.09, *SE* = 0.47, *p* = 0.021) perceivers, and the suburban relation was not significant (*B* = 0.69, *p* = 0.221). Figure 2 displays two illustrative contrasting categories and the conditional slope for every category with its confidence interval and sample size.

As exploratory quantities that depend on the serial mediation specification, the conditional serial indirect relations followed the same pattern (very rural: −0.33, 95% CI [−0.83, 0.07]; urban: 0.21, 95% CI [0.05, 0.44]; major metropolitan: 0.39, 95% CI [0.15, 0.73]), and the index of moderated mediation excluded zero (0.18, 95% CI [0.05, 0.36]). Because the indirect paths themselves are fragile (Section 4.4), we treat these conditional estimates as exploratory rather than as evidence of a moderated caregiving process. We do not interpret any single moderator value as a substantive boundary, and non-significance in the sparse rural-leaning categories should not be read as evidence of no relation in those groups.

### 4.4. Sensitivity Analyses

Diagnostics supported the moderation, and they also showed that the indirect decomposition is fragile. Variance inflation factors for the core predictors were at most 2.5, indicating that the burden–ambivalence correlation did not destabilize estimation. The interaction was unchanged with HC3 heteroscedasticity-robust standard errors (*p* = 0.004) and after excluding 35 influential cases (Cook’s distance > 4/*N*; *B* = 0.54, *p* = 0.002). A Harman single-factor test indicated that the first factor explained 36.3% of item variance, below the conventional 50% threshold. We report this as a standard screen rather than as evidence of absence. The test is widely regarded as insensitive, and common-method variance cannot be ruled out without a longitudinal design or a measured marker variable, neither of which is available here [59]. In a parallel specification, the indirect relation through burden was negative with an interval that included zero (−0.19, 95% CI [−0.43, 0.01]), the relation through ambivalence was positive (0.19, 95% CI [0.02, 0.45]), and a reversed serial ordering produced a non-significant indirect relation (−0.10, 95% CI [−0.26, 0.01]). These sign changes across specifications are why we decline to give the indirect paths substantive interpretation.

Five sample-focused checks addressed covariates and composition. With education, household income, employment status, and co-residence added (*N* = 416), the interaction held (*B* = 0.61, *p* = 0.003), as it did within the co-residing subsample (*n* = 336; *B* = 0.52, *p* = 0.022) and within the plausibility-screened subsample (*n* = 368; *B* = 0.67, *p* = 0.002; index of moderated mediation = 0.14, 95% CI [0.02, 0.30]). It also held when the 25 analytic-sample participants who failed the unenforced attention check were excluded (*n* = 399; *B* = 0.75, *SE* = 0.21, *p* = 0.0005). Replacing the perceived measure with the objective RUCA metropolitan versus nonmetropolitan indicator produced no significant interaction (*B* = 0.26, *SE* = 0.79, *p* = 0.745; 43 nonmetropolitan participants), so the moderation is specific to perceived community type in these data, whether because perception is what matters or because the objective test lacked power. Finally, because the outcome takes only nine observed values and concentrates at the ceiling, we re-estimated the two focal associations with a proportional-odds (ordered logistic) specification treating those nine values as ordinal categories. Both held. Greater strain was associated with lower odds of a higher quality of life category (odds ratio = 0.62 per strain point, *p* < 0.001), the ambivalence × area type interaction remained (*b* = 0.07, *p* = 0.004; categorical joint likelihood-ratio test χ^2^(4) = 20.25, *p* < 0.001), and the conditional slopes kept the same signs and ordering as the linear model. Neither focal result therefore appears to depend on treating the 0–100 metric as continuous, though the outcome’s coarseness remains a limitation.

## 5. Discussion

This study examined whether perceived economic strain was associated with caregiving grandparents’ quality of life through caregiver burden and caregiver ambivalence, and whether perceived residential area type moderated the ambivalence–quality of life relation. Two qualifications frame everything that follows. First, during data verification, we identified and corrected a storage-code error in the health-satisfaction item of the outcome measure, and all results reflect the corrected scoring. Second, the design is cross-sectional, all measures are same-source self-reports, and the moderator is a single ordinal item, so the models describe conditional associations rather than processes.

For RQ1, the data supported the component associations but not the hypothesized pathway. Greater strain was associated with more endorsed burdens (H1a), and burden with greater ambivalence (H1b). However, ambivalence was positively associated with quality of life once strain and burden were controlled, contrary to H1c, and the serial indirect relation was positive rather than negative, contrary to H1d. The dominant finding for RQ1 is simpler. Perceived economic strain showed a strong, direct negative relation with quality of life (standardized total effect = −0.49), consistent with the financial-distress literature [14,18,20], and the appraisal pathway carried almost none of it. We caution against reading the positive adjusted ambivalence coefficient as a benefit of ambivalence. Given the 0.75 burden–ambivalence correlation and the sign instability across parallel and reversed specifications, the adjusted coefficients for the two appraisals are best read as unstable decompositions of overlapping constructs [61].

For RQ2, the moderation was supported and robust within these data. The relation between ambivalence and quality of life differed across perceived residential area type. It was negative among small-town perceivers, directionally negative among very rural perceivers, and positive among urban and major metropolitan perceivers. The pattern held with expanded covariates, robust standard errors, influence checks, the co-residing subsample, and the plausibility screen, and it did not replicate with an objective metropolitan versus nonmetropolitan indicator. These results are consistent with the idea that the meaning of ambivalent feelings for well-being depends on the community context caregivers perceive themselves to live in [8]. They are also consistent with subtler possibilities, including composition differences among people who describe the same kinds of places differently.

Why might ambivalence relate to lower well-being among small-town perceivers and higher well-being among metropolitan perceivers? We did not measure any candidate mechanism, so we offer possibilities only as hypotheses for future research. Small communities can involve close, overlapping networks in which conflicted feelings toward family may be harder to hold [6,62]. Anonymity or normalized service use in metropolitan settings might make ambivalence less costly. Among urban perceivers, ambivalence may also accompany engaged, high-contact caregiving that is otherwise experienced as rewarding [63,64]. Testing these ideas requires measuring stigma, network overlap, service use, and caregiving rewards directly.

The study makes three contributions. First, it provides what is, to our knowledge, the first test of whether perceived residential area type moderates the relation between a caregiving appraisal and quality of life among caregiving grandparents, and the moderation it identifies is robust across covariate sets, robust standard errors, influence checks, subsamples, and an ordinal specification of the outcome. Second, because the dataset pairs perceived place with an objective RUCA classification, the study can show that the two behave differently in the same sample, which echoes calls to treat them as distinct constructs [8] and is a concrete reason for future studies to measure both. Third, it applies the ABC-X framework to caregiving grandparents without forcing the moderator into the B factor, offering a cleaner way to place community context in family stress models. We still do not claim the study extends the model’s theory. The B factor was not measured, and the ordering of the C-factor appraisals could not be established.

Implications for policy and practice are preliminary. The strongest and most consistent finding is the negative relation between perceived economic strain and quality of life (standardized total effect = −0.49), which held in every specification we tested. This supports sustained attention to financial hardship in policies and programs that serve caregiving grandparents, consistent with existing recommendations [14,20]. Additionally, the moderation suggests, if it replicates, that the same ambivalent feelings may carry different meaning in different perceived community contexts. Practitioners who work with caregiving grandparents should not assume that conflicted feelings about caregiving signal distress in the same way for every family, and intervention research should measure perceived community context directly. We do not recommend screening for ambivalence on the basis of these data. No validated grandparent-specific tool, threshold, or evidence of benefit exists, and screening for a sensitive feeling could cause harm if implemented carelessly.

The strengths of this study include the verification of every reported value against the raw data, with the scoring correction identified during that verification and disclosed transparently, hypotheses specified in advance of these analyses (although after data collection, and without public preregistration) and evaluated as stated against the corrected results, categorical and continuous treatments of the moderator, and a thorough set of diagnostics and robustness checks. The dataset also pairs perceived and objective place classifications, which is what made the perceived-versus-objective comparison possible.

Several limitations bound what these findings can support. The cross-sectional, same-source design cannot order the constructs in time, and common-method variance may inflate associations [58,59]. The Harman test we report is an insensitive screen, and ruling out method variance would require a longitudinal design or a marker variable that this survey did not include. The outcome is a two-item global facet with nine observed score values and a ceiling-heavy distribution, so linear regression on it approximates a coarse variable. The moderator is a single ordinal self-classification whose categories mix settlement size, density, and identity. Its two rural-leaning categories contain 65 participants combined, so the negative conditional relations there are imprecise, and the moderation did not replicate with a coarse objective indicator. The burden count is dichotomized and overlaps heavily with ambivalence, which destabilizes their unique coefficients. The sample is an opt-in online panel that is more affluent, more educated, more male-oriented, and more urban-perceiving than the custodial grandparent population that motivates much of this study; eligibility required daily contact but set no minimum number of care hours; and complete-case exclusions, though few, assume ignorable missingness. Finally, the sample is likely least representative of grandparents facing the greatest rural service barriers, which is where the negative ambivalence relations appeared.

## 6. Conclusions

Among 424 caregiving grandparents from an opt-in online panel, perceived economic strain showed a strong negative relation with quality of life, and the relation between caregiver ambivalence and quality of life depended on perceived residential area type, negative among small-town perceivers and positive among urban and metropolitan perceivers. The hypothesized serial appraisal pathway was not supported. These findings are exploratory associations in a nonrepresentative sample, and they do not demonstrate that perceived place causes differences in well-being. They are most useful as a guide for future research, especially in the following two areas: Perceived economic strain showed a strong, robust relation with quality of life in every specification we tested, which supports keeping the financial circumstances of caregiving grandparents on the policy agenda. Additionally, perceived community context, measured with a single inexpensive item, moderated an appraisal-to-well-being relation that an objective classification did not, which makes how caregivers see their own communities a promising measure to carry into future intervention research. That research should measure community context directly (e.g., service access, transportation, social networks, stigma, and community resources), follow families over time, and test whether the perceived-place moderation replicates in samples with substantial rural representation.

## Figures and Tables

**Table 1:** Participant demographic information.

	*n*	%
Ethnicity		
Hispanic or Latinx	47	11.08
Not Hispanic or Latinx	377	88.92
Race		
White	368	86.79
Black or African American	27	6.37
Native Hawaiian or Pacific Islander	2	0.47
Asian	1	0.24
American Indian or Alaska Native	1	0.24
Two or more races	13	3.07
Race not reported	12	2.83
Sex		
Male	220	51.89
Female	203	47.88
Not listed	1	0.24
Age Category		
35–44 years old	115	27.12
45–54 years old	133	31.37
55–64 years old	107	25.24
Over 65 years old	69	16.27
Household Income		
<$25,000	25	5.90
$25,000–<$50,000	64	15.09
$50,000–<$75,000	36	8.49
$75,000–<$100,000	41	9.67
$100,000–<$150,000	133	31.37
≥$150,000	124	29.25
Declined to respond	1	0.24
Education Level Obtained		
No High School Degree	2	0.47
High School Diploma or Equivalency (GED)	69	16.27
Some College	1	0.24
Associate’s Degree or Vocational Degree/License	40	9.43
Bachelor’s Degree	75	17.69
Master’s Degree	168	39.62
Doctorate or Professional Degree	69	16.27
Work Status		
Employed, Full-Time	274	64.62
Employed, Part-Time	18	4.25
Retired	103	24.29
Unemployed/Homemaker	22	5.19
Other	7	1.65

Note: Frequencies and valid percentages are for the analytic sample (*N* = 424). Race and ethnicity were asked in a single check-all-that-apply item that listed Hispanic or Latinx among the race options. Ethnicity is recovered from that option and race from the remaining options. Twelve participants selected only Hispanic or Latinx and are shown as race not reported. Because the item did not use the two-question format recommended for these constructs, race among Hispanic or Latinx participants is likely undercounted. Income category boundaries follow the survey’s response options, with each boundary value belonging to the lower category. Participants who selected “None of the above” for education and provided a text response were recoded based on that response: two reported not completing high school, one reported some college without a degree, and one reported trade school (recoded to the associate or vocational category).

**Table 2:** Descriptive statistics for primary study variables.

Variable	*N*	Minimum	Maximum	*M*	*SD*	Possible Range
Perceived Economic Strain (IFDFW, Reverse-Coded)	424	1.00	10.00	3.83	2.15	1–10
Quality of Life (WHOQOL-BREF General Facet, Corrected)	424	0.00	100.00	78.80	20.41	0–100
Caregiver Burden (CBS Count)	424	0.00	13.00	4.93	4.60	0–13
Caregiver Ambivalence (CAS Total Score)	424	0.00	15.00	4.78	4.52	0–15
Perceived Residential Area Type	424	1.00	5.00	3.55	1.08	1–5

Note. Descriptive statistics are based on the analytic sample (*N* = 424) with the corrected quality of life scoring.

**Table 3:** Bivariate correlations among primary study variables and covariates (*N* = 424).

	1	2	3	4	5	6	7	8
1. Perceived Economic Strain (ES)	—							
2. Quality of Life (QoL)	−0.58 ***	—						
3. Caregiver Burden (CB)	0.03	0.04	—					
4. Caregiver Ambivalence (CA)	−0.06	0.17 ***	0.75 ***	—				
5. Perceived Area Type (PA)	−0.39 ***	0.34 ***	0.26 ***	0.34 ***	—			
6. Age	0.21 ***	−0.24 ***	−0.24 ***	−0.25 ***	−0.31 ***	—		
7. Female	0.31 ***	−0.28 ***	−0.16 ***	−0.23 ***	−0.31 ***	0.22 ***	—	
8. Married	−0.40 ***	0.38 ***	0.16 ***	0.29 ***	0.31 ***	−0.30 ***	−0.37 ***	—

Note. *N* = 424 (analytic sample), corrected quality-of-life scoring. ES = economic strain; QoL = quality of life; CB = caregiver burden; CA = caregiver ambivalence; PA = perceived residential area type (higher = more urban perception). Female = female (1) versus male (0), computed for the 423 participants who selected male or female. Married = married (1) versus not married (0). *** *p* < 0.001.

**Table 4:** Results of serial mediation and moderated serial mediation models predicting quality of life.

Predictor	Outcome	*B*	β	*SE*	*t*	*p*	95% LLCI	95% ULCI
**Model 6 Equation for Caregiver Burden (*R*^2^ = 0.11)**								
Economic Strain (X)	Burden (M1)	0.398	0.186	0.114	3.50	0.0005	0.174	0.621
Age	Burden (M1)	−0.090	−0.228	0.020	−4.58	<0.001	−0.129	−0.052
Female (vs. Male)	Burden (M1)	−1.216	—	0.476	−2.55	0.0110	−2.152	−0.280
Widowed (vs. Married)	Burden (M1)	−0.377	—	0.758	−0.50	0.6193	−1.867	1.113
Divorced (vs. Married)	Burden (M1)	−1.435	—	0.868	−1.65	0.0992	−3.141	0.272
Single, Never Married (vs. Married)	Burden (M1)	−2.867	—	1.058	−2.71	0.0070	−4.946	−0.788
Other Marital Status (vs. Married)	Burden (M1)	−3.717	—	2.623	−1.42	0.1571	−8.873	1.438
**Model 6 Equation for Caregiver Ambivalence (*R*^2^ = 0.59)**								
Economic Strain (X)	Ambivalence (M2)	−0.011	−0.005	0.077	−0.14	0.8892	−0.162	0.140
Burden (M1)	Ambivalence (M2)	0.694	0.707	0.033	21.22	<0.001	0.630	0.759
Age	Ambivalence (M2)	−0.010	−0.027	0.013	−0.77	0.4435	−0.037	0.016
Female (vs. Male)	Ambivalence (M2)	−0.517	—	0.320	−1.62	0.1065	−1.146	0.111
Widowed (vs. Married)	Ambivalence (M2)	−1.286	—	0.505	−2.54	0.0113	−2.279	−0.292
Divorced (vs. Married)	Ambivalence (M2)	−1.598	—	0.580	−2.75	0.0061	−2.740	−0.457
Single, Never Married (vs. Married)	Ambivalence (M2)	−1.773	—	0.711	−2.49	0.0130	−3.171	−0.375
Other Marital Status (vs. Married)	Ambivalence (M2)	−0.556	—	1.752	−0.32	0.7510	−4.001	2.888
**Model 6 Equation for Quality of Life (*R*^2^ = 0.38)**								
Economic Strain (X)	QoL (Y)	−4.643	−0.490	0.427	−10.88	<0.001	−5.481	−3.804
Burden (M1)	QoL (Y)	−0.482	−0.109	0.262	−1.84	0.0670	−0.998	0.034
Ambivalence (M2)	QoL (Y)	0.718	0.159	0.273	2.63	0.0088	0.181	1.254
Age	QoL (Y)	−0.132	−0.075	0.075	−1.77	0.0774	−0.279	0.015
Female (vs. Male)	QoL (Y)	−2.102	—	1.781	−1.18	0.2386	−5.603	1.399
Widowed (vs. Married)	QoL (Y)	−5.982	—	2.827	−2.12	0.0350	−11.539	−0.424
Divorced (vs. Married)	QoL (Y)	−4.412	—	3.252	−1.36	0.1756	−10.804	1.980
Single, Never Married (vs. Married)	QoL (Y)	−4.928	—	3.978	−1.24	0.2161	−12.748	2.891
Other Marital Status (vs. Married)	QoL (Y)	−5.234	—	9.728	−0.54	0.5909	−24.357	13.890
Total Effect of X	QoL (Y)	−4.644	−0.490	0.423	−10.98	<0.001	−5.475	−3.813
Serial Indirect (X → M1 → M2 → Y), Bootstrap	QoL (Y)	0.198	0.021	—	—	—	0.040	0.410
**Model 87 Equation for Quality of Life (*R*^2^ = 0.40)**								
Economic Strain (X)	QoL (Y)	−4.298	−0.453	0.444	−9.68	<0.001	−5.171	−3.425
Burden (M1)	QoL (Y)	−0.547	−0.123	0.260	−2.11	0.0358	−1.058	−0.037
Ambivalence (M2)	QoL (Y)	−1.765	−0.391	0.824	−2.14	0.0328	−3.384	−0.145
Perceived Area Type (W)	QoL (Y)	−0.588	−0.031	1.079	−0.55	0.5860	−2.709	1.533
M2 × W Interaction	QoL (Y)	0.632	—	0.203	3.11	0.0020	0.232	1.032
Age	QoL (Y)	−0.118	−0.067	0.075	−1.57	0.1164	−0.265	0.029
Female (vs. Male)	QoL (Y)	−2.016	—	1.776	−1.14	0.2569	−5.508	1.475
Widowed (vs. Married)	QoL (Y)	−5.813	—	2.793	−2.08	0.0380	−11.303	−0.323
Divorced (vs. Married)	QoL (Y)	−4.467	—	3.213	−1.39	0.1653	−10.783	1.850
Single, Never Married (vs. Married)	QoL (Y)	−5.042	—	3.947	−1.28	0.2023	−12.801	2.718
Other Marital Status (vs. Married)	QoL (Y)	−6.044	—	9.619	−0.63	0.5301	−24.952	12.863
Index of Moderated Mediation, Bootstrap	QoL (Y)	0.180	—	—	—	—	0.045	0.359

Note. *N* = 424, corrected quality-of-life scoring. *B* = unstandardized coefficient; β = standardized coefficient (not shown for categorical indicators). Marital status enters every model as indicator variables with married as the reference category, and sex as a female versus male indicator. All models also include an indicator for the one participant who selected a not-listed sex option. That coefficient is estimated from a single case and is omitted from the table. Boot CIs are percentile bootstrap intervals (5000 resamples). Total effect is the relation between strain and quality of life controlling only for covariates.

## Data Availability

The raw data supporting the conclusions of this article will be made available by the authors on request.

## Supplementary Materials

The following supporting information can be downloaded at https://www.mdpi.com/article/10.3390/healthcare14182972/s1. Table S1: Response-option audit trail for the WHOQOL-BREF health-satisfaction item.
