# Supplementary material for: It Takes a Village (or a City): Perceived Economic Strain, Caregiver Ambivalence, and Quality of Life Among Caregiving Grandparents Across Perceived Residential Area Types
Source: Healthcare (Basel). 2026 Sep 11;14(18):2972. doi: 10.3390/healthcare14182972 (PMC13606181; doi:10.3390/healthcare14182972)
Supplement: Supplementary file 1 [file healthcare-14-02972-s001.zip › healthcare-4486936-supplementary.pdf]

Supplementary Materials

Table S1. Response-option audit trail for the WHOQOL-BREF health-satisfaction item ("How satisfied are you with your health?").

Response option	Stored code in the export	Value assigned by the earlier recode	Corrected value	n (delivered dataset, N = 439)
Very dissatisfied	1	1	1	9
Dissatisfied	3	3	2	39
Neither satisfied nor dissatisfied	4	4	3	40
Satisfied	5	5	4	202
Very satisfied	7	2	5	149

Note. Stored codes are the Qualtrics option identifiers in the raw survey export. The five response options were administered as ordinary satisfaction anchors. In the export metadata, the first option's label contains a typographical error ("Very dissatisified"). The earlier recode reassigned only the stored code 7, mapping it to scale point 2, which scored the 149 most-satisfied respondents as dissatisfied and left the stored codes 3, 4, and 5 one scale point too high. The corrected mapping assigns the five stored codes to scale points 1 through 5 by position. The corrected item was checked for convergent plausibility against SF-12 physical and mental component scores, self-rated general health, and depressive symptoms, each of which changes monotonically across the corrected item. The corrected general facet correlates r = .29 with the previously analyzed version.
